# Symptom Profiles and Progression in Hospitalized and Nonhospitalized Patients with Coronavirus Disease, Colorado, USA, 2020

**DOI:** 10.3201/eid2702.203729

**Published:** 2021-02

**Authors:** Grace M. Vahey, Kristen E. Marshall, Emily McDonald, Stacey W. Martin, Jacqueline E. Tate, Claire M. Midgley, Marie E. Killerby, Breanna Kawasaki, Rachel K. Herlihy, Nisha B. Alden, J. Erin Staples, on behalf of the Colorado Investigation Team

**Affiliations:** Centers for Disease Control and Prevention, Fort Collins, Colorado, USA (G.M. Vahey, E. McDonald, S.W. Martin, J.E. Staples);; Centers for Disease Control and Prevention, Atlanta, Georgia, USA (K.E. Marshall, J.E. Tate, C.M. Midgley, M.E. Killerby);; Colorado Department of Public Health and Environment, Denver, Colorado, USA (K.E. Marshall, B. Kawasaki, R.K. Herlihy, N.B. Alden)

**Keywords:** 2019 novel coronavirus disease, COVID-19, SARS-CoV-2, coronavirus disease, severe acute respiratory syndrome coronavirus 2, viruses, respiratory infections, zoonoses, symptoms, symptom progression, symptom duration, hospitalization

## Abstract

To improve recognition of coronavirus disease (COVID-19) and inform clinical and public health guidance, we randomly selected 600 COVID-19 case-patients in Colorado. A telephone questionnaire captured symptoms experienced, when symptoms occurred, and how long each lasted. Among 128 hospitalized patients, commonly reported symptoms included fever (84%), fatigue (83%), cough (73%), and dyspnea (72%). Among 236 nonhospitalized patients, commonly reported symptoms included fatigue (90%), fever (83%), cough (83%), and myalgia (74%). The most commonly reported initial symptoms were cough (21%–25%) and fever (20%–25%). In multivariable analysis, vomiting, dyspnea, altered mental status, dehydration, and wheezing were significantly associated with hospitalization, whereas rhinorrhea, headache, sore throat, and anosmia or ageusia were significantly associated with nonhospitalization. General symptoms and upper respiratory symptoms occurred earlier in disease, and anosmia, ageusia, lower respiratory symptoms, and gastrointestinal symptoms occurred later. Symptoms should be considered alongside other epidemiologic factors in clinical and public health decisions regarding potential COVID-19 cases.

Severe acute respiratory syndrome coronavirus 2 (SARS-CoV-2), the virus that causes coronavirus disease (COVID-19), was first detected in China in December 2019 (*1*,*2*). Within 1 month, COVID-19 cases were reported in numerous countries, including the United States (*3*). By the end of January 2020, the World Health Organization (WHO) declared the COVID-19 outbreak a public health emergency of international concern (*4*). After WHO’s declaration, rapid acceleration of virus transmission in many parts of the world led WHO to characterize COVID-19 as a global pandemic in March (*5*). As of December 4, the United States had reported >14 million COVID-19 cases and ≈275,000 associated deaths (*6*). The large number of cases and deaths has created an unprecedented burden on the nation’s healthcare system, necessitating triage of patients and the prioritization of testing.

Initially, the most common symptoms of COVID-19 were reported to be fever, cough, and dyspnea (*7*–*9*). However, asymptomatic infections and additional symptoms common to other viral respiratory illnesses have been reported, including chills, fatigue, myalgia, sore throat, nasal congestion, rhinorrhea, nausea, vomiting, and diarrhea (*10*). Persons with COVID-19 have also reported anosmia (loss of smell) and ageusia (loss of taste) more frequently than with other viral respiratory diseases (*11*).

Although ≈80% of persons with COVID-19 experience mild disease (*12*), to date most published reports of COVID-19 symptoms are derived from case-series and cross-sectional analyses of medical record reviews, primarily among hospitalized patients. Literature regarding symptoms experienced by nonhospitalized COVID-19 patients is growing, but information summarizing symptom duration, progression, and statistical comparison to hospitalized patients remains limited. To improve COVID-19 disease recognition, which can help mitigate its spread, particularly for mild cases, and inform clinical and public health guidance, we interviewed hospitalized and nonhospitalized COVID-19 patients in Colorado to determine the symptoms they experienced and when these symptoms occurred during their course of illness.

## Methods

### Sample

Hospitalized and nonhospitalized patients were identified from laboratory-confirmed COVID-19 cases reported to the Colorado Electronic Disease Reporting System (CEDRS) as of April 5, 2020. Based on data available in CEDRS, patients were considered eligible if they had known hospitalization status; had self-reported illness onset during March 9–31, 2020; and resided in 1 of the 9 counties (Adams, Arapahoe, Boulder, Denver, Douglas, El Paso, Jefferson, Larimer, and Weld) that account for ≈80% of Colorado’s population. March 9 was selected because it was the date on which testing for SARS-CoV-2 became more widely available in Colorado and was no longer restricted to suspected cases requiring hospitalization or having an epidemiologic link to a confirmed case, though travel to an area with ongoing community transmission was required for testing early in this period. To obtain interviews from at least 300 patients (200 nonhospitalized and 100 hospitalized), we used stratified, simple random sampling to select 600 patients (using a 2:1 ratio) from 1,738 COVID-19 cases meeting inclusion criteria.

### Data Collection

At least 3 attempts were made to contact each selected patient on at least 2 separate days, at different times of the day, during April 10–30, 2020. For contacted patients who consented, a trained public health official administered a standardized questionnaire by telephone to obtain demographic information, verify hospitalization status and date of illness onset, and determine whether the patient had experienced any of 30 symptoms during their illness. For patients whose hospitalization status differed between CEDRS data and interview, we confirmed status using electronic medical records. For all deceased patients, minors, and persons unable to be interviewed (e.g., those with dementia), a proxy (i.e., relative or caregiver) was interviewed. Patients were asked what their first and subsequent symptoms were, and for each reported symptom, when it occurred relative to onset of illness and how long it lasted. No follow-up contact was made once the questionnaire was completed.

### Statistical Analysis

Data were entered into a Research Electronic Data Capture database (*13*,*14*). Frequencies and percentages were calculated and stratified by hospitalization status. We calculated odds ratios (ORs), 95% CIs, and p values to identify COVID-19 symptoms associated with hospitalization. Multivariable logistic regression was conducted to construct a model examining association of all symptoms with hospitalization status, while adjusting for demographic variables associated with hospitalization for COVID-19 (i.e., male sex, age >65 years, and Hispanic ethnicity) (Appendix Table). A reduced multivariable model was constructed by using purposeful selection to identify a subset of symptoms from the full model that had statistically significant association (*15*). In multivariable models, anosmia and ageusia were combined because of a high degree of collinearity; no other significant collinearity was identified.

Median and interquartile ranges (IQRs) were calculated for duration and timing of individual symptoms in relation to overall onset of illness. To account for patients who died and the large proportion of patients who were still symptomatic at the time of interview, we used survival analysis to calculate estimated median illness duration compared by hospitalization status. For participants still experiencing symptoms at interview, individual symptom duration was truncated to the date of interview because a low proportion (<10%) of patients reported individual symptoms still occurring at that time. Symptoms were categorized by organ system based on codes from the International Classification of Diseases, 10th Revision, Clinical Modification. Statistical analyses were conducted by using SAS 9.4 (SAS Institute, https://www.sas.com) and R version 3.6.3 software (https://r-project.org) (*16*). Significance was defined as α = 0.05, and all testing was 2-sided.

### Ethics Considerations

This investigation received a nonresearch determination as a public health response from human subjects advisors at the Centers for Disease Control and Prevention. The investigation was considered a public health response to a notifiable disease by the Colorado Department of Public Health and Environment.

## Results

### The Patients

Of 600 randomly selected case-patients, 364 (61%) completed the interview, 46 (8%) were ineligible (because onset date was before March 9 or they were asymptomatic), 57 (10%) declined to participate, and 133 (22%) were unreachable. Median age of the 364 participating patients was 50 years (range 2 months–94 years); 187 (51%) were male, 288 (79%) identified as white, and 75 (21%) identified as Hispanic. Almost all patients (345 [95%]) reported having health insurance; 128 (35%) patients were hospitalized, and 18 (5%) died. Compared with nonhospitalized patients, hospitalized patients were older and more likely to be Black; they were also more likely to be male (Table 1). Compared with patients who declined to participate or were unreachable, investigation participants resided proportionately in the same counties and had similar hospitalization rates (35% vs. 31%) and case-fatality ratios (5% vs. 8%), but they were older than nonparticipating patients (median age 50 vs. 43 years).

**Table 1:** Demographics, interview information, hospitalization status, and outcome of 364 patients with laboratory-confirmed coronavirus disease by hospitalization status, Colorado, USA, March 2020

Characteristic	No. (%)	
	Hospitalized, n = 128	Nonhospitalized, n = 236
Sex		
M	79 (62)	108 (46)
F	49 (38)	127 (54)
Other	0 (0)	1 (<1)
Age group, y		
<18	3 (2)	1 (<1)
19–44	23 (18)	118 (50)
45–64	50 (39)	84 (36)
>65	52 (41)	33 (14)
Race*		
White	95 (74)	193 (82)
Black	13 (10)	12 (5)
Asian	9 (7)	9 (4)
Pacific Islander	1 (1)	3 (1)
American Indian	2 (2)	2 (1)
Other	9 (7)	18 (8)
Unknown	4 (3)	4 (2)
Ethnicity		
Non-Hispanic or Latino	86 (67)	163 (69)
Hispanic or Latino	29 (23)	46 (19)
Unknown	13 (10)	27 (11)
Health insurance status and type		
Insured*	118 (92)	227 (96)
Private	64 (50)	184 (78)
Medicare	41 (32)	21 (9)
Medicaid	22 (17)	13 (6)
Military or Tricare	5 (4)	11 (5)
Not specified	1 (1)	2 (1)
Not insured	8 (6)	6 (3)
Unknown	2 (2)	3 (1)
Interview type		
Patient interview	96 (75)	226 (96)
Proxy interview	32 (25)	10 (4)
Outcome		
Survived	113 (88)	233 (99)
Died	15 (12)	3 (1)
*Options were not mutually exclusive.		

Among 364 participating patients, interviews were conducted with 322 (88%) patients and proxies for 42 (12%) patients. Patients who were interviewed directly reported a higher median number of symptoms (13 [IQR 9–16]) than proxies reported (6 [IQR 4–10]). Proxies were interviewed more frequently for hospitalized patients than for nonhospitalized patients (Table 1) and more often for participants >65 years of age (30/85 [35%]) than for those <65 years (12/279 [4%]). Median number of days from illness onset to interview (33 days) did not differ by hospitalization status.

### Frequency of Symptoms

Based on International Classification of Diseases, Tenth Revision, Clinical Modification, categorization of symptoms, general systemic symptoms (i.e., fever, chills, myalgia, headache, or anorexia) were commonly reported among both hospitalized (122 [95%]) and nonhospitalized (234 [99%]) patients (Table 2). Symptoms associated with potential lower respiratory tract infection (cough, dyspnea, wheezing, or chest pain) were reported by 116 (91%) of hospitalized patients and 213 (90%) of nonhospitalized patients. Cognition and perception symptoms (altered mental status, anosmia, or ageusia) were reported by 87 (68%) hospitalized and 165 (70%) nonhospitalized patients. More nonhospitalized patients (158 [67%]) reported upper respiratory tract infection symptoms (i.e., rhinorrhea, nasal congestion, or sore throat) than were reported by hospitalized patients (60 [47%]). Gastrointestinal symptoms (i.e., nausea, vomiting, diarrhea, or abdominal pain) were reported by 58% of participants, regardless of hospitalization status.

**Table 2:** Frequency and duration of symptoms reported by 364 hospitalized and nonhospitalized patients with laboratory-confirmed coronavirus disease, Colorado, USA, March 2020*

Symptoms	Hospitalized, n = 128			Nonhospitalized, n = 236		Crude OR (95% CI)	p value
	No. (%)	Median symptom duration (IQR)		No. (%)	Median symptom duration (IQR)		
Symptom groups							
Any general symptom†	122 (95)	NC		234 (99)	NC	NC	NC
Any LRI symptom‡	116 (91)	NC		213 (90)	NC	NC	NC
Any cognitive or perception symptom§	87 (68)	NC		165 (70)	NC	NC	NC
Any URI symptom¶	60 (47)	NC		158 (67)	NC	NC	NC
Any GI symptom#	74 (58)	NC		136 (58)	NC	NC	NC
Individual symptoms							
Fever**	108 (84)	**		196 (83)	**	1.10 (0.61–2.01)	0.74
Fatigue	106 (83)	14 (9–27)		213 (90)	12 (7–15)	0.52 (0.28–0.98)	0.04
Any cough††	93 (73)	NC		196 (83)	NC	NC	NC
Dry cough	79 (62)	10 (7–22)		175 (74)	10 (5–18)	0.56 (0.35–0.89)	0.01
Chills	84 (66)	7 (3–10)		169 (72)	3 (2–7)	0.76 (0.48–1.20)	0.24
Myalgia	72 (56)	11 (7–15)		175 (74)	5 (3–9)	0.45 (0.28–0.71)	<0.01
Anorexia	89 (70)	12 (7–17)		150 (64)	7 (4–11)	1.31 (0.83–2.09)	0.25
Dyspnea	92 (72)	10 (5–19)		144 (61)	10 (6–14)	1.63 (1.03–2.62)	0.04
Headache	66 (52)	8 (4–14)		166 (70)	7 (3–14)	0.45 (0.29–0.70)	<0.01
Ageusia	63 (49)	14 (8–21)		149 (63)	10 (7–20)	0.57 (0.37–0.87)	0.01
Sweats	70 (55)	7 (3–10)		134 (57)	3 (2–7)	0.92 (0.60–1.42)	0.70
Anosmia	45 (35)	14 (7–24)		131 (56)	10 (7–21)	0.43 (0.28–0.67)	<0.01
Diarrhea	60 (47)	7 (3–13)		104 (44)	3 (2–6)	1.12 (0.73–1.73)	0.61
Arthralgia	45 (35)	13 (7–17)		100 (42)	5 (4–10)	0.74 (0.47–1.15)	0.18
Dehydration	54 (42)	10 (4–14)		76 (32)	5 (3–10)	1.54 (0.98–2.40)	0.06
Chest pain	42 (33)	10 (5–16)		85 (36)	7 (4–14)	0.87 (0.55–1.36)	0.54
Rhinorrhea	31 (24)	7 (3–12)		97 (41)	7 (4–14)	0.46 (0.28–0.73)	<0.01
Sore throat	28 (22)	8 (4–15)		91 (39)	4 (2–7)	0.45 (0.27–0.72)	<0.01
Nasal congestion	28 (22)	7 (3–14)		86 (36)	7 (5–14)	0.49 (0.29–0.79)	<0.01
Nausea	41 (32)	7 (3–12)		69 (29)	4 (2–7)	1.14 (0.71–1.81)	0.58
Wheezing	44 (34)	12 (5–16)		54 (23)	9 (6–14)	1.77 (1.10–2.84)	0.02
Productive cough	37 (29)	10 (7–28)		58 (25)	10 (5–16)	1.25 (0.77–2.02)	0.37
Altered mental status	39 (30)	7 (3–16)		39 (17)	6 (3–12)	2.21 (1.33–3.69)	<0.01
Abdominal pain	18 (14)	9 (7–20)		49 (21)	3 (2–5)	0.62 (0.34–1.11)	0.12
Conjunctivitis	16 (13)	7 (3–12)		36 (15)	5 (3–10)	0.79 (0.41–1.47)	0.47
Vomiting	24 (19)	4 (2–6)		24 (10)	2 (1–4)	2.04 (1.10–3.77)	0.02
Lymphadenopathy	7 (5)	7 (6–13)		37 (16)	6 (3–10)	0.31 (0.12–0.68)	<0.01
Rash	9 (7)	4 (2–7)		24 (10)	5 (3–10)	0.67 (0.29–1.44)	0.32
Hemoptysis	8 (6)	7 (4–9)		7 (3)	3 (3–9)	2.18 (0.77–6.36)	0.14
Seizures	3 (2)	7 (4–10)		0	NC	NC	NC

*GI, gastrointestinal; IQR, interquartile range; LRI, lower respiratory tract infection; NC, not calculated; OR, odds ratio; URI, upper respiratory tract infection.  †General symptoms included fever, chills, sweats, myalgia, headache, fatigue, arthralgia, dehydration, anorexia, and lymphadenopathy.  ‡ LRI symptoms included cough (dry and productive), dyspnea, wheezing, hemoptysis, and chest pain.  §Cognition and perception symptoms included anosmia, ageusia, and altered mental status.  ¶URI symptoms included nasal congestion, rhinorrhea, and sore throat. #GI symptoms included nausea, vomiting, diarrhea, and abdominal pain. **Fever was collected individually as subjective or measured. Values were combined given potential bias because hospitalized patients were more likely to have their temperature measured compared with nonhospitalized patients, who more commonly reported subjective fever only. The median duration of both subjective and measured fevers in nonhospitalized patients was 4 d (IQR 2–7 d). In hospitalized patients, the median duration of measured fever was 7 d [IQR 3–11 d] and subjective fever was 8 d (IQR 4–13 d). ††Any cough is a combination of dry cough, productive cough, and hemoptysis, which are also reported individually.

The most frequently reported symptoms were similar for hospitalized and nonhospitalized participants (Table 2). Among 128 hospitalized patients, the most commonly reported symptoms were fever (108 [84%]), fatigue (106 [83%]), cough (93 [73%]), and dyspnea (92 [72%]). Among 236 nonhospitalized patients, the most commonly reported symptoms were fatigue (213 [90%]), fever (196 [83%]), cough (196 [83%]), and myalgia (175 [74%]). Ageusia was reported by 149 (63%) nonhospitalized and 63 (49%) hospitalized patients, and anosmia by 131 (56%) nonhospitalized and 45 (35%) hospitalized patients. A total of 123 (96%) hospitalized patients and 229 (97%) nonhospitalized patients reported fever, cough, or dyspnea. Of the 12 participants not reporting these symptoms, the most commonly reported symptoms were fatigue (7 patients), anosmia (6 patients), ageusia (6 patients), anorexia (6 patients), and diarrhea (5 patients).

Participants who reported altered mental status and vomiting had at least twice the odds of being hospitalized (Table 2). Patients reporting wheezing and dyspnea also had higher odds of hospitalization. In contrast, patients who reported lymphadenopathy, anosmia, rhinorrhea, myalgia, headache, sore throat, or nasal congestion had less than half the odds of hospitalization. Patients reporting fatigue, dry cough, and ageusia also had lower odds of hospitalization.

**Figure 1:**
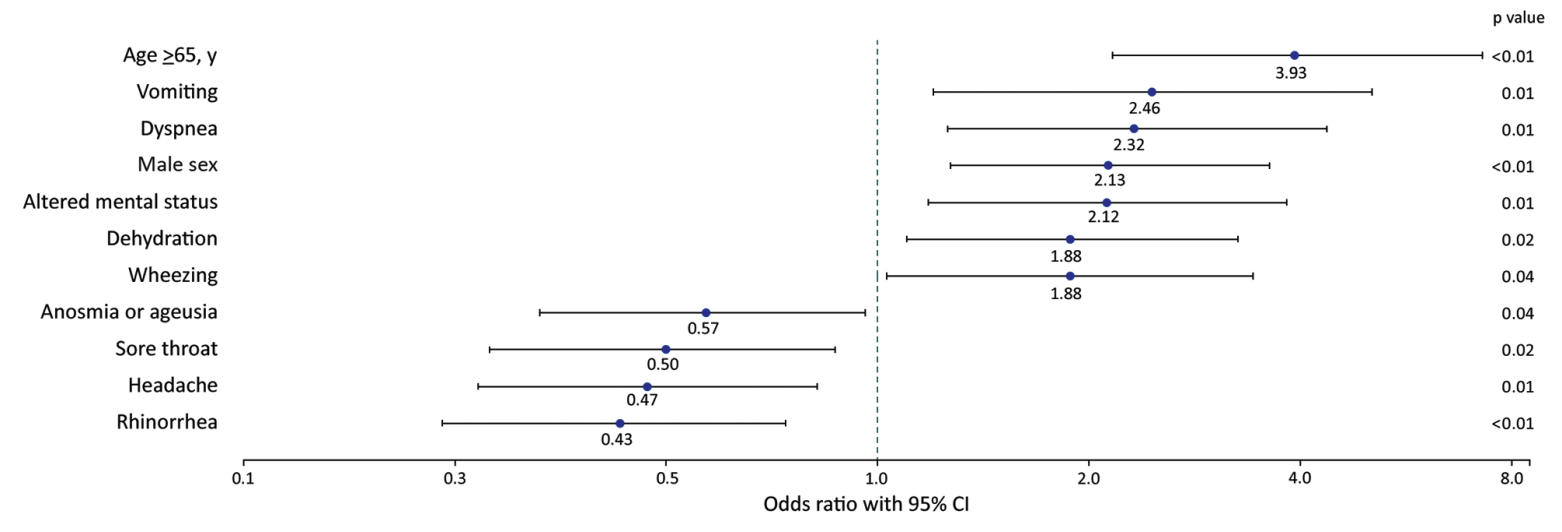
Coronavirus disease symptoms significantly associated with hospitalization in reduced multivariable model (n = 364 patients), Colorado, March 2020.

When we controlled for all reported symptoms and characteristics included in the reduced multivariable logistic regression model, we found that participants who reported vomiting (OR 2.46 [95% CI 1.2–5.06]), dyspnea (OR 2.32 [95% CI 1.26–4.37]), altered mental status (OR 2.12 [95% CI 1.18–3.83]), dehydration (OR 1.88 [95% CI 1.1–3.26]), and wheezing (OR 1.88 [95% CI 1.03–3.43]) had higher odds of hospitalization, as did participants who were male (OR 2.13 [95% CI 1.27–3.62]) or >65 years of age (OR 3.93 [95% CI 2.16–7.27]) (Figure 1). Patients reporting rhinorrhea (OR 0.43 [95% CI 0.24–0.74]), headache (OR 0.47 [95% CI 0.27–0.82]), sore throat (OR 0.5 [95% CI 0.28–0.87]), and anosmia or ageusia (OR 0.57 [95% CI 0.33–0.96]) had lower odds of hospitalization.

### Temporal Occurrence of Symptoms

The most common initial symptoms for hospitalized and nonhospitalized patients were cough (25% for hospitalized and 21% for nonhospitalized patients) and fever (25% for hospitalized and 20% for nonhospitalized patients) (Table 3). No participants reported conjunctivitis, rash, or lymphadenopathy as an initial symptom of their illness. Patients reporting sore throat as their initial symptom had lower odds of being hospitalized (OR 0.28 [95% CI 0.11–0.74]); no other initial symptom was associated with hospitalization status.

**Table 3:** Initial symptom reported by 364 hospitalized and nonhospitalized patients with laboratory-confirmed coronavirus disease, Colorado, USA, March 2020*

Symptom	No. (%)	
	Hospitalized, n = 128	Nonhospitalized, n = 236
Cough	32 (25)	49 (21)
Fever	32 (25)	47 (20)
Fatigue	17 (13)	44 (19)
Headache	14 (11)	45 (19)
Myalgia	14 (11)	38 (16)
Sore throat†	5 (4)	30 (13)
Chills	11 (9)	19 (8)
Nasal congestion	2 (2)	12 (5)
Dyspnea	8 (6)	6 (3)
Ageusia	1 (1)	7 (3)
Diarrhea	2 (2)	6 (3)
Anosmia	1 (1)	6 (3)
Rhinorrhea	2 (2)	5 (2)
Chest pain	1 (1)	5 (2)
Abdominal pain	3 (2)	2 (1)
Altered mental status	4 (3)	1 (<1)
Sweats	2 (2)	2 (1)
Wheezing	1 (1)	2 (1)
Vomiting	2 (2)	1 (<1)
Dehydration	1 (1)	1 (<1)
Anorexia	1 (1)	1 (<1)
Nausea	0	1 (<1)
Seizures	1 (1)	0
Conjunctivitis	0	0
Rash	0	0
Lymphadenopathy	0	0

*Reported symptoms are not mutually exclusive. †Indicates statistical significance with nonhospitalization.

**Figure 2:**
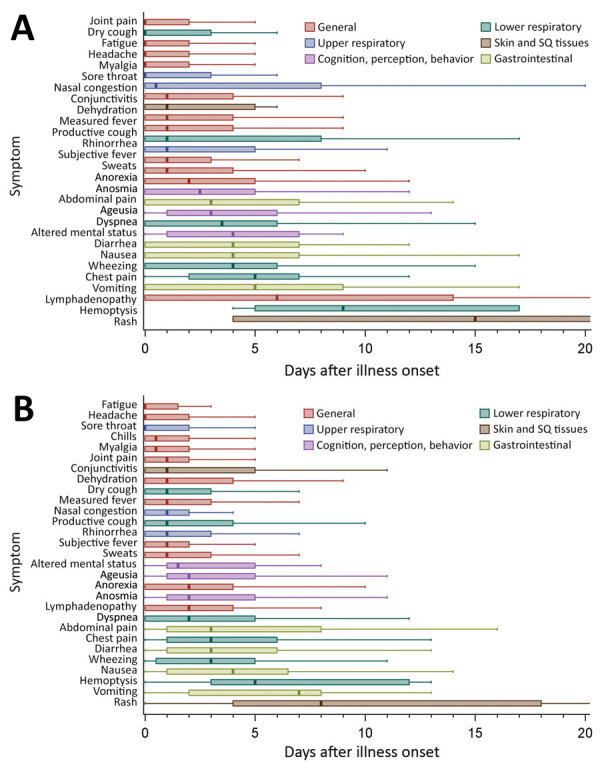
Days from coronavirus disease onset to individual symptom onset, by hospitalization status (n = 364 patients), Colorado, March 2020. Symptom progression is shown for hospitalized patients (A) and nonhospitalized patients (B). Lines within boxes indicate median for each symptom, and boxes represent interquartile range. Outliers (defined as >1.5× interquartile range >75th percentile) not shown in figure. SQ, subcutaneous.

Little variation was observed between hospitalized and nonhospitalized patients in terms of symptom progression (Figure 2). Upper respiratory symptoms and general systemic symptoms were reported early in the course of disease; many patients reported these types of symptoms within 1 day of illness onset. Symptoms related to cognition, perception, and lower respiratory tract (except cough) were generally reported to occur 2–4 days after illness onset. Gastrointestinal symptoms were reported to occur ≈3–6 days after illness onset, and rash generally appeared last.

Among 346 surviving patients, 134 (39%) were still symptomatic at time of interview. The estimated median duration of illness was 18 days longer in hospitalized patients (36 days; p<0.01) than in patients who were not hospitalized (18 days; p<0.01) (Appendix Figure). The median duration of most individual symptoms was <10 days; notable exceptions were fatigue for both hospitalized (14 days [IQR 9–27 days]) and nonhospitalized (12 days [IQR 7–15 days]) participants and, among hospitalized patients, anosmia (14 days [IQR 7–24 days]), ageusia (14 days [IQR 8–21 days]), arthralgia (13 days [IQR 7–17 days]), anorexia (12 days [IQR 7–17 days]), wheezing (12 days [IQR 5–16 days]), and myalgia (11 days [IQR 7–15 days]) (Table 2). The median durations of chills, myalgia, sweats, diarrhea, arthralgia, dehydration, sore throat, abdominal pain, vomiting, and hemoptysis for hospitalized patients were >2 times those of nonhospitalized patients.

## Discussion

We found that persons with COVID-19 in Colorado commonly reported fever, cough, or dyspnea, similar to findings in previous reports (*7*–*9*,*17*). However, we also identified several other symptoms (i.e., fatigue, chills, myalgia, anorexia, and headache) that occurred with similar frequency, and we noted differences in the frequency of symptoms reported by hospitalized and nonhospitalized participants.

In general, we found higher frequencies of symptoms than previously reported (*18*–*21*). This discrepancy is likely in part a result of our approach of collecting symptom data through standardized interviews compared with other reports that are based on data extracted from medical records. Data taken from medical records generally capture the most prominent symptoms reported when a patient seeks care and might not capture initial nonspecific symptoms or symptoms that occur later in the course of illness. For example, a medical chart review of 242 hospitalized patients with symptomatic COVID-19 in China found the most common symptoms at admission were fever (90%), cough (38%), and fatigue (16%), compared with rates of fever (84%), cough (73%), and fatigue (83%) in the hospitalized participants in our analysis (*18*). However, the higher frequencies of certain symptoms in our analysis might also be because of differences in the populations studied and their disease severity. For instance, the frequency of ageusia and anosmia among nonhospitalized patients in this analysis was similar to previous reports of patients with mild COVID-19 (*22*–*26*) but was higher than a smaller cohort of hospitalized patients in another study (*19*).

Patients in our cohort reported high frequencies of general symptoms and lower respiratory tract symptoms, including cough. More than half of our patients reported >1 gastrointestinal symptom regardless of hospitalization status, which was similar to findings from previous reports examining symptoms through interviews with hospitalized and nonhospitalized patients (*17*,*26*). The rates of gastrointestinal symptoms in this analysis are higher than a previous report that found 35% of persons receiving outpatient care for COVID-19 had diarrhea, nausea, or vomiting documented in their charts (*27*) and another study in which 19% of hospitalized COVID-19 patients had chart-documented diarrhea or abdominal pain at admission (*28*). One explanation for the differences in reported gastrointestinal symptoms is that these symptoms occur later in illness and might be absent when the patient initially seeks care. This progression was documented recently in a prospective investigation of nonhospitalized COVID-19 patients, in which only 23% of patients reported gastrointestinal symptoms at the time of their first positive SARS-CoV-2 test but 53% of all patients experienced gastrointestinal symptoms at some point in their illness (*22*). Other studies have found patients with gastrointestinal symptoms were more likely to seek medical care >1 week after onset of illness, compared with those without gastrointestinal symptoms, who were more likely to seek care <1 week after illness onset (*27*,*28*).

When comparing the frequency of reported symptoms between hospitalized and nonhospitalized patients, we found that patients reporting certain lower respiratory symptoms (wheezing and dyspnea), altered mental status, vomiting, and dehydration had higher odds of hospitalization. This finding is not surprising, because many of these symptoms would likely prompt a clinician to recommend inpatient management. Similarly, in a convenience sample of symptomatic persons with COVID-19 from 16 US states, dyspnea was more commonly reported by hospitalized patients, and anosmia, ageusia, and rhinorrhea were more commonly reported by nonhospitalized patients (*17*). Among all symptoms we associated with hospitalization, only dyspnea has been statistically associated with more serious disease, as measured by intensive-care unit admission (*29*).

A notable finding from our analysis was that upper respiratory tract symptoms were more commonly reported by nonhospitalized patients. This finding could aid in clinicians’ recognition of less severe disease and therefore help mitigate the spread of infection. Other nonspecific symptoms reported very commonly or rarely (namely, fatigue, dry cough, myalgia, and lymphadenopathy) were no longer significantly associated with nonhospitalization on multivariable analysis. Our findings among nonhospitalized patients are consistent with recent reports from Europe, South Korea, and the United States that found that upper respiratory symptoms, such as nasal congestion and rhinorrhea, were common among persons with mild or moderate COVID-19 (*22*,*30*,*31*). These findings suggest that potential differences in route of infection (i.e., contact with respiratory droplets vs. inhalation of aerosolized viral particles) could be related to the pathogenesis and severity of COVID-19, although other factors also likely contribute, such as age, underlying medical conditions, and viral strain. These findings also support the concept that COVID-19 manifests in 1 of 3 general patterns of illness: mild illness primarily consisting of upper respiratory symptoms, non–life-threatening pneumonia, and severe pneumonia with acute respiratory distress syndrome (*32*).

We found the most commonly reported initial symptoms for COVID-19 patients were cough or fever. These symptoms were also the most common initial symptoms reported by 48 healthcare personnel with COVID-19 in King County in Washington state (*33*). However, no single symptom was reported by more than one quarter of our participating patients as their initial symptom, suggesting the absence of a hallmark symptom at the beginning of disease.

In regards to symptom progression over the course of illness, upper respiratory symptoms, general systemic symptoms, and cough were reported to have occurred early in illness. These symptoms were followed by other lower respiratory symptoms, altered mental status, anosmia, ageusia, and, finally, gastrointestinal symptoms and rash. The timing of anosmia and ageusia in our analysis is similar to previous reports, which found a mean of 3 days from illness onset to anosmia and ageusia in hospitalized and nonhospitalized COVID-19 patients (*34*,*35*). The later occurrence of gastrointestinal symptoms and rash among our participants could be related directly to the virus, linked to interventions (e.g., use of antimicrobial drugs or other medications), or, in the case of gastrointestinal symptoms, related to hypoxia (*36*–*39*). We identified an overall progression of reported symptoms that is consistent with, although more detailed than, a recent metaanalysis of symptoms among persons with COVID-19 (*20*). In addition, symptom onset and progression in this investigation is similar to what has been described for severe acute respiratory syndrome (SARS), caused by SARS-CoV (*40*,*41*). SARS has been described to manifest with an initial phase of fever, cough, sore throat, and myalgia, followed by dyspnea, hypoxia, and diarrhea, and, in some patients, a final phase of acute respiratory distress syndrome (*42*).

In our investigation, the median duration of most symptoms was <10 days. However, estimated duration of illness was >1 month in hospitalized patients, twice as long as in nonhospitalized patients; this pattern was also observed for many individual symptoms. Duration of individual symptoms experienced by nonhospitalized patients was slightly longer in our analysis than in 2 previous reports of nonhospitalized COVID-19 patients; however, the symptoms with the longest duration were similar (cough, anosmia, and ageusia) and methods differed slightly between analyses (*24*,*43*). A report on symptoms experienced by nonhospitalized COVID-19 patients in Utah found a median duration of symptoms of 16 days, which is similar to our findings for nonhospitalized patients (*22*). Published data on COVID-19 symptoms in 2 studies of hospitalized patients in China found that fever duration was substantially longer in those with more severe disease (*18*,*44*).

Our investigation has some limitations. First, interviews were conducted several weeks after illness onset, which enabled accurate classification of patients by hospitalization status and data collection on all symptoms and their duration (*45*). However, this timing might result in incomplete recall and recall bias, which could affect the accuracy of reported symptoms and their timing, particularly among hospitalized patients, who might be more likely to remember more severe symptoms (*46*). Future prospective studies using methods such as symptom diaries or serial interviews could reduce recall bias. Second, a higher proportion of proxies were interviewed on behalf of hospitalized case-patients. However, when proxies were removed from the reduced multivariable model, the ORs were relatively stable, indicating the proxies did not affect the association of symptoms with hospitalization. In addition, although clinical manifestation of viral respiratory diseases can differ by age, we were unable to compare symptoms across different age groups because of the high percentage of proxy interviews for patients >65 years of age, which resulted in fewer symptoms being reported in that age group. Our findings might not apply to all populations because of differences in age distribution, disease severity, testing practices, and socioeconomic status. Finally, because symptoms such as seizure and hemoptysis were experienced by a small number of participants, we were limited in our ability to draw conclusions about their duration and associations with hospitalization status.

Overall, in this study, patients with COVID-19 commonly reported fever, cough, or dyspnea. However, other symptoms occurred frequently, less than one quarter of participants reported any 1 individual symptom as their initial symptom, and the frequency of symptoms reported by hospitalized and nonhospitalized patients was notably different. A person’s symptoms should be considered alongside local disease prevalence and other epidemiologic factors (e.g., age, underlying conditions, and exposures to known and suspected COVID-19 cases) for clinical decision-making, such as testing and differential diagnosis, and for determining appropriate public health action for persons with potential COVID-19. Clinicians should consider COVID-19 in addition to other common respiratory pathogens in patients with mild or nonspecific symptoms to help mitigate the spread of the disease. Furthermore, public health messaging should continue to encourage social distancing, use of masks, and good hand hygiene for everyone and self-isolation for anyone with potential COVID-19 symptoms.

Appendix Additional information about symptom profiles and progression in hospitalized and nonhospitalized patients with coronavirus disease, Colorado, USA, 2020.
