# Supplementary material for: Symptom Profiles and Progression in Hospitalized and Nonhospitalized Patients with Coronavirus Disease, Colorado, USA, 2020
Source: Emerg Infect Dis. 2021 Feb;27(2):385–95. doi: 10.3201/eid2702.203729 (PMC7853576; doi:10.3201/eid2702.203729)
Supplement: Appendix — Additional information about symptom profiles and progression in hospitalized and nonhospitalized patients with coronavirus disease, Colorado, USA, 2020. [file 20-3729-Techapp-s1.pdf]

Article DOI: https://doi.org/10.3201/eid2702.203729

Symptom Profiles and Progression in Hospitalized and Nonhospitalized Patients with Coronavirus Disease, Colorado, USA, 2020

Appendix

Appendix Table. Full multivariable model of symptom association with hospitalization status in 364 patients with coronavirus disease, Colorado, USA, March 2020*

Category	OR	SE	95% CI	p value
Age ≥65 y	3.23	0.34	1.68–6.32	<0.01
Sex, M	1.94	0.44	0.82–4.60	.13
Hispanic ethnicity	1.14	0.36	0.56–2.32	.72
Dyspnea	2.68	0.34	1.38–5.34	<0.01
Altered mental status	2.29	0.29	1.31–4.10	<0.01
Dehydration	2.15	0.32	1.14–4.08	.02
Vomiting	1.73	0.3	0.97–3.11	.06
Anorexia	1.66	0.35	0.85–3.31	.14
Wheezing	1.62	0.68	0.42–6.32	.48
Hemoptysis	1.58	0.33	0.84–3.01	.16
Sweats	1.44	0.34	0.74–2.79	.28
Diarrhea	1.32	0.32	0.72–2.47	.37
Arthralgia	1.31	0.32	0.70–2.47	.4
Productive cough	1.27	0.32	0.68–2.41	.46
Subjective or objective fever	1.25	0.31	0.68–2.29	.48
Chest pain	1.14	0.41	0.51–2.62	.75
Nasal congestion	1.06	0.35	0.53–2.10	.86
Nausea	1.03	0.34	0.53–1.99	.93
Rash	0.97	0.48	0.36–2.39	.94
Conjunctivitis	0.93	0.39	0.42–1.98	.85
Chills	0.86	0.37	0.42–1.76	.67
Abdominal pain	0.79	0.39	0.36–1.69	.55
Dry cough	0.77	0.31	0.42–1.41	.39
Sore throat	0.55	0.34	0.28–1.07	.08
Headache	0.53	0.33	0.28–1.01	.05
Myalgia	0.5	0.32	0.26–0.92	.03
Anosmia or ageusia	0.5	0.31	0.27–0.91	.02
Fatigue	0.46	0.48	0.18–1.17	.1
Rhinorrhea	0.39	0.33	0.20–0.74	<0.01
Lymphadenopathy	0.38	0.53	0.13–1.04	.07

*OR, odds ratio; SE, standard error.

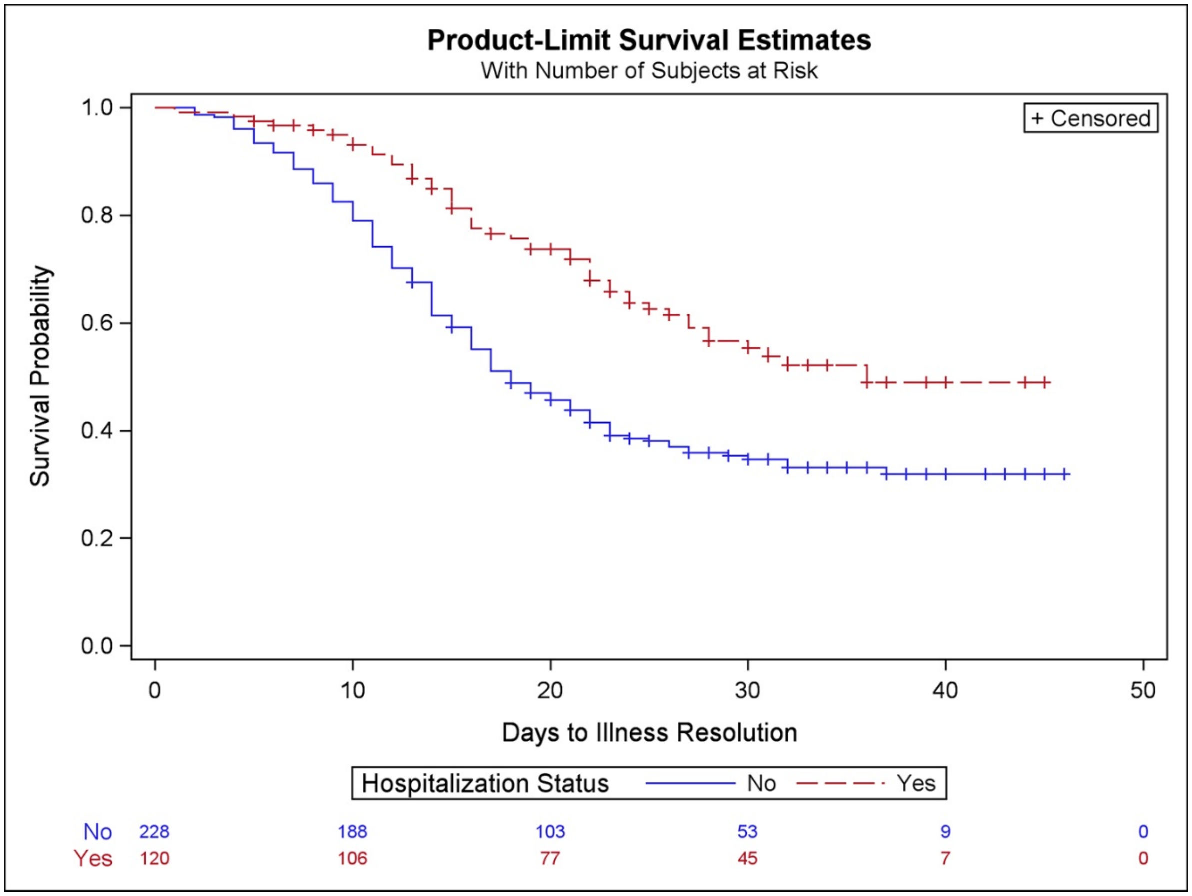

Appendix Figure. Survival analysis of illness duration by hospitalization status in patients with laboratory-confirmed coronavirus disease (n = 364), Colorado, March 2020. Observations were censored (indicated by a plus symbol) if the patient died or was symptomatic at the time of interview.
